# Cardamom, Cumin, and Dill Weed Essential Oils: Chemical Compositions, Antimicrobial Activities, and Mechanisms of Action against *Campylobacter* spp.

**DOI:** 10.3390/molecules22071191

**Published:** 2017-07-15

**Authors:** Aysegul Mutlu-Ingok, Funda Karbancioglu-Guler

**Affiliations:** Department of Food Engineering, Faculty of Chemical and Metallurgical Engineering, Istanbul Technical University, Maslak, 34469 Istanbul, Turkey; mutluays@itu.edu.tr

**Keywords:** antimicrobials, cell constituents’ release, extracellular ATP concentration, relative electric conductivity

## Abstract

Natural antimicrobials as well as essential oils (EOs) have gained interest to inhibit pathogenic microorganisms and to control food borne diseases. *Campylobacter* spp. are one of the most common causative agents of gastroenteritis. In this study, cardamom, cumin, and dill weed EOs were evaluated for their antibacterial activities against *Campylobacter jejuni* and *Campylobacter coli* by using agar-well diffusion and broth microdilution methods*,* along with the mechanisms of antimicrobial action. Chemical compositions of EOs were also tested by gas chromatography (GC) and gas chromatography-mass spectrometry (GC-MS). The results showed that cardamom and dill weed EOs possess greater antimicrobial activity than cumin with larger inhibition zones and lower minimum inhibitory concentrations. The permeability of cell membrane and cell membrane integrity were evaluated by determining relative electric conductivity and release of cell constituents into supernatant at 260 nm, respectively. Moreover, effect of EOs on the cell membrane of *Campylobacter* spp. was also investigated by measuring extracellular ATP concentration. Increase of relative electric conductivity, extracellular ATP concentration, and cell constituents’ release after treatment with EOs demonstrated that tested EOs affected the membrane integrity of *Campylobacter* spp. The results supported high efficiency of cardamom, cumin, and dill weed EOs to inhibit *Campylobacter* spp. by impairing the bacterial cell membrane.

## 1. Introduction

*Campylobacter* spp. are Gram negative bacteria considered as the most common cause of bacterial-mediated diarrhoeal disease and human gastroenteritis [1]. Among the *Campylobacter* spp., *Campylobacter jejuni* and *Campylobacter coli* are the most frequently reported in human diseases. Although investigations concerning *Campylobacter* infections in Turkey are relatively limited, in Europe it was reported that the incidence of campylobacteriosis was about 9.2 million cases in 2009 [2]. Moreover, these infections are dramatically increasing worldwide over the last 10 years [3]. They are also the most prevalent bacterial food-borne pathogens in the industry [4]. These organisms can be isolated from a variety of sources including animal and human feces, water, and various foods [5]. *C. jejuni* and *C. coli* were also frequently isolated from raw poultry meat and cause food poisoning in humans when undercooked products are consumed [6]. There are several conventional methods to control these microorganisms including the use of synthetic antimicrobials. Due to high level of antimicrobial resistance and concerns on the use of synthetic antimicrobials, different novel approaches are necessary to control corresponding microorganisms.

Utilization of essential oils (EOs) as an alternative to synthetic antimicrobials is an increasing trend nowadays. Plant EOs are aromatic oily liquids which can be obtained by expression, fermentation, enfleurage, extraction, or steam distillation from different parts of plants [7]. Although, commonly known with their flavoring properties, their antibacterial, antioxidant, and antifungal properties have recently been of great interest. It has been reported that the antimicrobial activity of EOs is generally due to phenolic and terpenoid compounds [8,9,10] as well as aliphatic compounds [11]. Medicinal plants including cardamom, cumin, and dill which belong to the families of Zingiberaceae, Apiaceae, and Umbelliferae, respectively, have beneficial effects especially against human diseases due to their bioactive compounds [12]. These medicinal plants can be found in different countries around the world. Mainly India and Guatemala are the cardamom growing countries [13]. Although cumin is naturally growing in northern Egypt, the Mediterranean region, Iran, and India, today it is also cultivated in Mediterranean countries, Saudi Arabia, Iran, India, Mexico, and China [14]. Dill is commercially cultivated in most parts of Europe, although the native source of it is south-east Europe [15]. Essential oils formed as secondary metabolites of these plants have been widely used for thousands of years. In recent decades particularly, stronger antioxidant [16] and more fungitoxic potential [17] have been reported in comparison with synthetic ones for cumin EO. Antiaflatoxigenic effects of cardamom [18] and cytotoxic effects of dill EO [19] were also clarified. For wide-range properties of cardamom, cumin, and dill EOs, investigation of their chemistry, bioactivity, and action mode is quite important.

Despite the high number of studies on the antimicrobial effects of EOs, most studies have focused on pathogenic bacteria like *Staphylococcus aureus*, *Escherichia coli,* and *Bacillus cereus*. Moreover, little work has been reported on the mechanism of action of EOs. In these limited studies, antimicrobial activity mechanism of EOs against microorganisms were mainly evaluated by damage to the integrity of cell membrane [10,20,21,22], by leakage of electrolytes [22,23], and the loss of cell constituents [10,22]. To our knowledge, there are no studies reported on the mechanism of antimicrobial action of cardamom, cumin, and dill weed against *Campylobacter* spp. Therefore, the objectives of the present study were (i) to investigate the chemical compositions and antimicrobial activities of cardamom, cumin and dill weed EOs on *Campylobacter* spp. and (ii) to determine the mechanism of action responsible for antimicrobial activity by relative electric conductivity, cell constituent’s release, and extracellular ATP concentration measurements.

## 2. Results

### 2.1. Chemical Compositions of EOs

The chemical compositions of cardamom, cumin, and dill weed EOs were analyzed by GC and GC-MS. Table 1 shows the chemical components of tested EOs which were present in amount more than 0.5%. α-Pinene, the monoterpene hydrocarbon, was present in tested EOs with relatively low concentrations. The main constituents of EOs were *p*-mentha-1,3-dien-7-al (26.7%), cumin aldehyde (24.1%), γ-terpinene (16.9%), and β-pinene (14.4%) in cumin; α-terpinly acetate (43.4%) and 1,8-cineole (29.2%) in cardamom; carvone (41.6%) and limonene (27.4%) in dill weed EOs.

### 2.2. Antimicrobial Activity of Essential Oils

Initial screening of antimicrobial activity of the cardamom, cumin, and dill weed EOs was studied against *Campylobacter* spp. using an agar-well diffusion assay by measuring the diameter of the inhibition zone (DIZ). After that, minimum inhibition concentration (MIC) and minimum bactericide concentration (MBC) values were determined. As shown in Table 2, tested EOs displayed a variable degree of antimicrobial activity. For both *C. jejuni* and *C. coli* isolates, antimicrobial effects of cardamom, cumin, and dill weed EOs were statistically different from each other in terms of DIZ values (*p* < 0.05). Using agar-well diffusion assay, all EOs inhibited the growth of tested bacteria at different levels. The maximum DIZ value was obtained for cardamom essential oil, followed by dill weed and cumin EOs. Obtained MIC values confirmed the results of agar-well diffusion assay. Cumin EO had the highest MIC value (0.05 (µL/mL) against both *C. jejuni* and *C. coli*.

### 2.3. Relative Electric Conductivity (REC)

**Figure 1:**
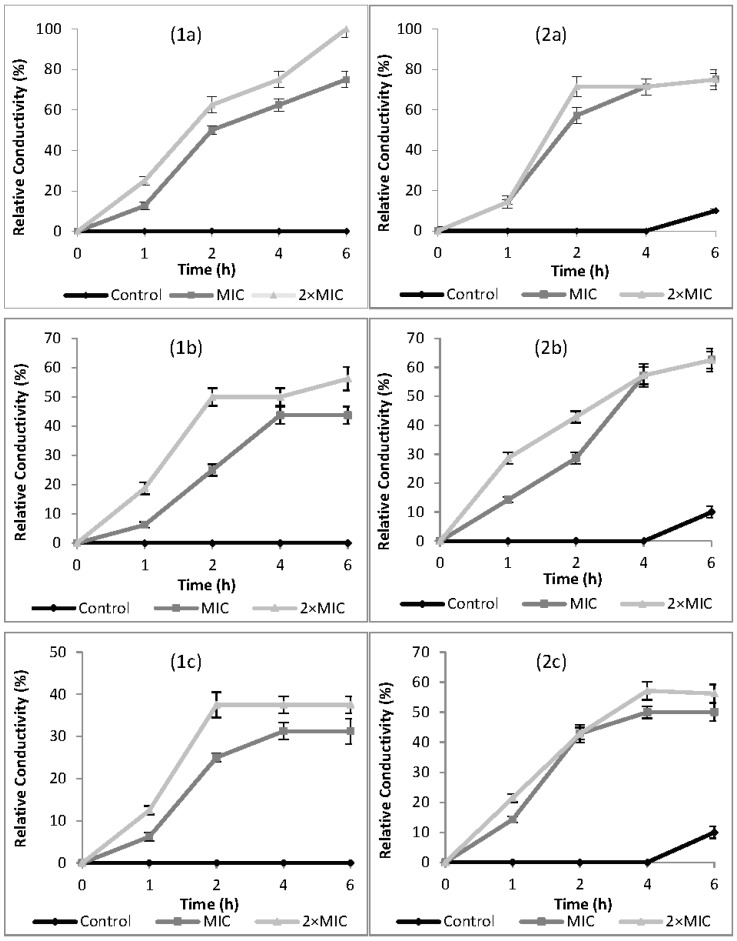
Effect of cardamom (**a**), cumin (**b**), and dill weed (**c**) essential oils on cell membrane permeability of *Campylobacter jejuni* (1) and *Campylobacter coli* (2), MIC: Minimum inhibition concentration.

The effects of cardamom, cumin, and dill weed EOs on cell membrane permeability of *Campylobacter* spp. are presented in terms of relative electric conductivity (REC) in Figure 1. The relative electric conductivity of the suspension would be visibly increased with increasing EO concentration and contact time. Electrical conductivity results showed that the membrane permeability of tested bacteria was increased when exposed to MIC and 2× MIC concentrations of EOs. The results were in accordance with the agar-well diffusion and broth microdilution results against *Campylobacter* spp. For all EOs, an increase of REC was observed in the first 4 h of incubation period. There was a little change from 4 h to 6 h of incubation period.

### 2.4. Cell Constituents’ Release

Cell membrane integrity was investigated by determination of released cell constituents, such as nucleic acids, proteins, and metabolites from *Campylobacter* spp. by measuring the absorbance of the supernatant at 260 nm. Results are shown in Table 3 when *C. jejuni* and *C. coli* were treated with cardamom, cumin, and dill weed EOs. The OD_260_ values at MIC and 2× MIC concentrations were significantly different from control and each other (*p* < 0.05). The maximum cell constituents’ release was observed after adding cumin and dill weed EOs against *C. jejuni* at 2× MIC concentration. Concentrations of cell constituents increased 2.2 to 5.3 times when treated with MIC concentrations, while they increased 2.8 to 9.1 when treated with 2× MIC concentrations of EOs compared to control.

### 2.5. Extracellular ATP Concentrations

**Figure 2:**
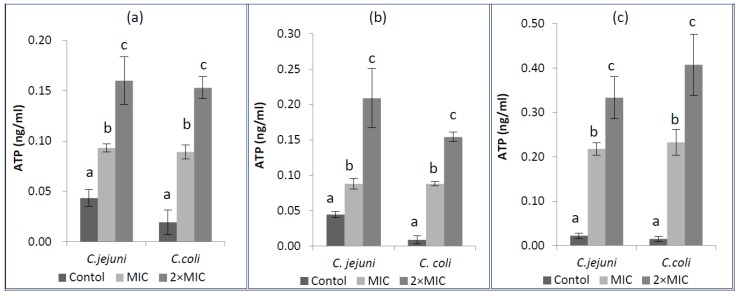
Extracellular ATP concentrations of *Campylobacter* spp. after adding cardamom (**a**), cumin (**b**), and dill weed (**c**) essential oils, MIC: Minimum inhibition concentration.

The mode of antimicrobial action was also observed by confirmation of extracellular ATP concentrations when tested microorganisms were exposed to EOs at their MIC and 2× MIC concentrations. Changes in extracellular ATP concentrations for *C. jejuni* and *C. coli* after exposure to EOs are shown in Figure 2. The values in the untreated *C. jejuni* and *C. coli* varied from 0.022 to 0.044 ng/mL for *C. jejuni* and 0.009 to 0.019 ng/mL for *C. coli*. The level of extracellular ATP of *Campylobacter* spp. increased significantly as EO concentration increased (*p* < 0.05). The increase of extracellular ATP concentrations when *Campylobacter* spp. were exposed to EOs indicates that tested EOs caused release of ATP from intracellular to extracellular medium.

## 3. Discussion

EOs and derived compounds have variety of biological properties; in addition to their antibacterial activities, they show antifungal [29], antiviral [30], antioxidant [31], antitumor activities [32], and mycotoxin inactivation characteristic [33,34]. Biological activities of EOs are related with their chemical compositions [35]. The results of GC-MS analysis indicated that chemical composition profiles obtained for EOs were very similar to the previous results of different researchers with minor differences. In cumin EO, cumin aldehyde (36%) has been reported as the major component [36]. In contrast, in the present study *p*-mentha-1,3-dien-7-al was the major component, followed by cumin aldehyde. The differences in the contents may be a result of differences in the geographical origin of the plant, use of different parts of plants, extraction method, and season of harvest [7]. The chemical composition profile of cardamom EO confirms previous studies where the main components were α-terpinyl acetate and 1,8-cineole [37,38]. Dill weed EO is predominantly composed of carvone (41.6%) and limonene (27.4%), followed by dill ether (9.2%). Current study results were in accordance with previous reports [39,40].

The results of antimicrobial activity tests indicated that cardamom, cumin, and dill weed EOs had high antimicrobial activity against *Campylobacter* spp. similar to other pathogenic bacteria listed in the literature. Research on the antimicrobial effects of EOs on *Campylobacter* spp. are mainly focused on thyme and oregano EOs [41,42]. EOs tested in the current study were only studied by Friedman et al. [43] who showed that in case of bactericidal activities, cardamom was the most active essential oil against *C. jejuni,* similar to our results. In the present study, based on antimicrobial test results, *Campylobacter* spp. had different levels of susceptibility to tested EOs. DIZ values were in the range of 19.75 ± 2.70–24.75 ± 2.00 and 21.08 ± 1.38–25.58 ± 2.23 for *C. jejuni* and *C. coli*, respectively. The MIC and MBC values were in the range of 0.012–0.05 µL/mL. MIC and MBC values were the same for all tested EOs. This phenomenon was also approved by El Bouzidi et al. [44], in which the MBC values were reported to be the same as the MIC values as investigated by the macrodilution method. Equivalent MIC and MBC values were also detected by Diao et al. [22] for fennel seed EO against *S. typhimurium*. The main constituents of EOs, including carbohydrates, alcohols, ethers, aldehydes, and ketones are responsible for their biological properties [45]. These major components present in tested EOs might also be related with their antimicrobial activity against *Campylobacter* spp. In the current study, the high content of carvone and limonene perhaps played an important role for high level of antimicrobial activity of dill weed EO. It is also stated by Delaquis et al. [39] that tested strains were inhibited by carvone-rich fractions of dill, but it is slightly less effective than d-limonene. High antifungal activity of carvone was also stated against *Sclerotinia sclerotiorum* [46]. Similar to the results of present study, cardamom EO which was rich of α-terpinyl acetate and 1,8 cineole, possesses high antibacterial properties against foodborne and medically important bacteria [47]. In terms of the antimicrobial activity rank of EO components, aldehydes are the second crucial group following phenols [45]. Possibly due to high level of cumin aldehyde content, a group of aldehydes, the antimicrobial activity level of cumin was significant [48]. High antimicrobial activity of cumin seed EO with cumin aldehyde(36.0%) was also reported [36].

In addition to antibacterial activities, the antimicrobial activity mechanism of EOs against pathogenic microorganisms should also be clarified. To the best of our knowledge, the mode of action has not been evaluated for *Campylobacter* spp. in great detail. The previous reports mainly focused on *E. coli*, *S. aureus*, and *B. cereus* and they reported that antimicrobial activity of EOs was mainly due to the disturbance of the cytoplasmic membrane and the proton motive force (PMF), electron flow, active transport, and coagulation of cell contents [7]. It is also reported that the toxic action of cyclic hydrocarbons to microorganisms is primarily via disruption of the cytoplasmic membrane [49].

In this study, the mechanism of antimicrobial action was confirmed according to the results of relative electric conductivity, release of cell constituents at 260 nm, and extracellular ATP concentrations when *Campylobacter* spp. were treated with MIC and 2× MIC concentrations of EOs. The antimicrobial action modes of EOs were firstly revealed by the permeability of the cell membrane based on relative electric conductivity measurements. RECs of suspensions rapidly increased after the addition of EOs with increasing contact time and concentration (Figure 1). Increase in relative conductivities clearly indicated that the bacterial cell membrane had become permeable at different levels after treatment with EOs. The value of relative conductivity at 2× MIC levels increased faster than at MIC levels. Particularly, cardamom EO increased membrane permeability of *C. jejuni* to 100% compared with the control, indicating a complete release of electrolytes outside the cell by cellular leakage. Increase in the membrane permeability of tested bacteria by exposing to EOs may be due to lysis and death of bacteria which caused the leakage of intracellular ingredients, especially losses of electrolytes including K^+^, Ca^2+^, Na^+^ [22]. The leakage might be caused by the interaction of antimicrobial substances and the cytoplasmic membrane [23]. Similar to our findings, increasing RECs have been reported by other researchers for *E. coli*, *S. aureus* [50], *Shigella dysenteriae* [22], *Ralstonia solanacearum* [51], and *B. cereus* [52].

Similar to REC values, a significant increase in the optical density at 260 nm was observed with the increased concentrations of cardamom, cumin, and dill weed EOs. Although there have been several targets of active compounds for inhibition, leakage of intracellular material was a general phenomenon [53]. Information on the cell constituent release reveals the cell membrane integrity [50]. The results indicated that addition of corresponding EOs led to leakage of the 260 nm-absorbing material from the cell membrane. The maximum cell constituent release was observed for cumin and dill weed EOs against *C. jejuni* at 2× MIC concentration. Loss of cell constituents like proteins and some essential molecules [22] indicates cytoplasmic membrane damage [10]. There are several studies on the disruption of cytoplasmic membrane and cell lysis due to the release of cellular contents on *S. aureus* [10,54,55], *Bacillus subtilis* [10,56], *Saccharomyces cerevisiae* [10,57], *E. coli* [10,23], *E. coli* O157:H7, *Salmonella typhi* [21], and *Shigella dysenteriae* [22]. Tested EOs destroyed the membrane integrity of *Campylobacter* spp. and resulted in the death of bacteria, which was also directly related with the cell membrane integrity [58] or the membrane permeability [59].

The mode of antimicrobial action was also confirmed on the basis of extracellular ATP concentrations. A significant increase in extracellular ATP concentrations on both *C. jejuni* and *C. coli* was detected when compared with the control, which may be due to membrane permeability destruction (*p* < 0.05). The highest level was observed when *C. coli* was treated with 2× MIC of dill weed EO (0.408 ng/mL), indicating the maximum release of ATP into extracellular medium. Decreased level of intracellular ATP and also increased level of extracellular ATP have been reported by Helendar et al. [60] for carvacrol and thymol. Moreover, increasing extracellular ATP concentrations have also been reported by other researchers [21,56,61]. Consistent with these studies, the increase in extracellular ATP levels in the current study indicates that tested EOs caused release of ATP out of cells. This situation could be related to envelope damage induced by the antimicrobial agents [21]. Depletion of ATP pools caused the impairment of essential processes in the cell and finally leads to cell death as ATP has several cellular functions that are necessary for growth, replication, and survival in living organisms [62,63].

Based on all results, cardamom and dill weed EOs showed higher antimicrobial activity against tested microorganisms compared to cumin EO, with larger DIZ and lower MIC values. REC, cell constituent release, and extracellular ATP concentration results demonstrated that cardamom, cumin, and dill weed EOs were effective antimicrobial agents targeting directly to the cell membrane of *Campylobacter* spp., disrupting the integrity and increasing the permeability, as well as causing loss of cellular material. Finally, these changes resulted in cell lysis and death of the bacterium.

## 4. Materials and Methods

### 4.1. Bacterial Culture and Essential Oils

The antimicrobial activity of the cold pressed cardamom (0.90 g/mL), cumin (0.93 g/mL), and dill weed (0.91 g/mL) EOs were tested against *Campylobacter jejuni* (ATCC 33660) and *Campylobacter coli* (NCTC 12525). EOs in food grade form were kindly provided by “International Flavors & Fragrances (IFF)”, Gebze, Kocaeli (Turkey). EOs without dilution and diluted in 10% dimethyl sulfoxide (DMSO, Merck, Darmsdat, Germany) were used in agar-well diffusion and broth micro dilution assays, respectively. EOs were sterilized before analysis by filtration through 0.22 µm filters and stored in the dark at 4 °C.

### 4.2. Gas Chromatography (GC)

Essential oils were analyzed by GC using an Agilent 7890B GC system with a flame ionization detector (FID). The chromatographic separation was accomplished using an Agilent HP-Innowax column (60 m × 0.25 mm Ø, with 0.25 µm film thickness) with a helium as a carrier gas (0.7 mL/min). GC oven temperature was kept as 60 °C for 10 min and programmed to 220 °C at a rate of 4 °C/min and then kept constant at 220 °C for 10 min and programmed to 240 °C at a rate of 1 °C/min. Split ratio was adjusted at 40:1. The injector and flame ionization detector temperatures were adjusted at 250 °C. The relative percentage amounts of the separated compounds were calculated from FID chromatograms.

### 4.3. Gas Chromatography-Mass Spectrometry (GC/MS)

The essential oils were analyzed by GC/MS using an Agilent 7890B GC coupled with a 5977B MSD (Agilent, Palo Alto, CA; SEM A. S., Istanbul, Turkey). The same column and analytical conditions were used for both GC/MS and GC/FID. The mass range was recorded from *m*/*z* 35 to 450. The injector temperature was adjusted at 250 °C. MS were recorded at 70 eV. Alkanes were used as reference points in the calculation of relative retention indices (RRI). The components of EOs were identified by using Wiley 9-Nist 11 Mass Spectral Database and standard Alkan series (C_7_–C_40_) and confirmed with the aid of retention indices from published sources.

### 4.4. Agar-Well Diffusion Assay

Diameter of inhibition zones (DIZ) was determined by the agar-well diffusion method [64]. Bacterial inoculum was prepared in Mueller-Hinton Broth (MHB, Merck, Darmsdat, Germany) and incubated at 42 °C for 48 h under microaerophilic conditions created by Anaerocult® C (Merck, Darmsdat, Germany). Concentrations of bacterial suspensions were adjusted to approximately 10^8^ CFU/mL and 100 µL of culture suspension was spread on modified CCDA medium (Merck, Darmsdat, Germany). Three wells were cut out of agar using a sterile cork borer and filled with 20 µL of EOs. The inoculated plates were incubated at 42 °C for 48 h under microaerophilic conditions. After incubation, DIZ values were measured with scale and recorded in mm. All experiments were performed in triplicate. Zones of inhibition (including the 6 mm of the well) were expressed as mean values with ± standard deviation.

### 4.5. Broth Microdilution Assay

To determine the MIC value, broth microdilution method was used which was described previously by Wiegand et al. [65]. Stock solutions of EOs were prepared in 10% DMSO and two-fold serial dilutions of EOs were made in the range of 0.003–30 µL/mL. After sub-culturing in MHB, bacterial concentration was adjusted to approximately 10^8^ CFU/mL. The 96-well plates were prepared by dispensing into each well 95 µL of MHB, 100 µL of EO, and 5 µL of the inoculants. The final volume in each well was 200 µL. The microplates were incubated at 42 °C for 24 h under microaerophilic conditions. MIC values were determined spectrophotometrically by measuring the optical density at an absorbance of 600 nm (Synergy HT, BioTek Instruments Inc., Winooski, VT, USA). Positive control was defined as wells containing inoculum but not EO, negative control was defined as wells containing EO but not inoculum. Minimum bactericidal concentration (MBC) was determined after MIC test. Broths from the wells (included MHB, isolates, and EOs) were cultured on modified CCDA and incubated at 42 °C for 48 h under microaerophilic conditions. MIC and MBC were the lowest bacteriostatic and bactericidal concentration of the tested EO under defined conditions, respectively.

### 4.6. Relative Electric Conductivity

Permeability of cell membrane was evaluated by determining relative electric conductivity as described by Kong et al. [23]. After sub-culturing in MHB, *Campylobacter* spp. were collected by centrifugation at 3000× *g* for 10 min and washed with 5% of glucose until their electric conductivities were close to that of 5% glucose, and they were the case for isotonic bacteria. Different concentrations of EOs (0 (control), MIC, 2× MIC) were added to 5% glucose and electric conductivities of the mixture were marked as *L*_1_. Different concentrations of EOs were added into the isotonic bacteria solution. After completely mixed, the samples were incubated at 42 °C, and then the conductivities were measured for 6 h and marked as *L*_2_. The control was the bacteria in 5% glucose treated in boiling water for 5 min and marked as *L*_0_. The ratio of % = 100 × (*L*_2_−*L*_1_)/*L*_0_ indicated as the REC. The bacterial cell membrane permeability was expressed with the ratio of REC.

### 4.7. Cell Constituents’ Release

The integrity of cell membrane of *Campylobacter* spp. was examined by the determination of cell constituents’ release into supernatant according to the method described by Rhayour et al. [66] with minor modifications. Cells from 50 mL of working culture of *Campylobacter* spp. (approximately 10^8^ CFU/mL) were collected by centrifugation (4000× *g* for 15 min), washed three times and resuspended in phosphate buffered saline (PBS, pH 7.4). 50 mL of cell suspensions were incubated at 42 °C for 4 h under agitation (IKA, KS, 4000 i control) in the presence of different concentrations of EOs (0 (control), MIC, 2× MIC). Then, 25 mL of samples were collected and centrifugated at 4000× *g* for 20 min. The concentration of the cell constituents in supernatant was determined by using UV-spectrophotometer at 260 nm. Correction was made with the same PBS containing the same concentration of EOs after 2 min contact with *Campylobacter* spp. The untreated cells (control) were corrected with PBS.

### 4.8. Extracellular ATP Determination

Extracellular ATP concentrations were measured using an ATP bioluminescent assay kit (Molecular probes, A22066) according to the method described by Lee et al. [67]. Culture cells were grown and a working culture (approximately 10^8^ CFU/mL) was centrifuged for 10 min at 1000× *g*. Cell pellets were washed with sodium phosphate buffer (0.1 M, pH 7.0) and then collected under the same conditions. Cell suspensions were prepared with 0.5 mL cell solution, 9 mL of sodium phosphate buffer (0.1 mol/L, pH 7), and different concentrations of EOs (0 (control), MIC, and 2× MIC). Samples were maintained at room temperature for 30 min, centrifuged for 5 min at 2000× *g*, and then incubated in ice bath to prevent ATP loss. Extracellular ATP concentrations of supernatant were determined by a luminometer (BioTek, Synergy HT) at 420 nm after addition of 100 µL of standard reaction solution into 10 µL of supernatant. Standard curve was prepared by using ATP standard solutions ranging from 0.01–1 nM. The regression (*y* = 1482*x* + 49.517, *r*^2^ = 0.9982) was obtained between ATP concentration (nM) and relative luminescence.

### 4.9. Statistical Analysis

All experiments were done in triplicate, and mean values were presented with ± standard deviation. One-way analysis of variance (ANOVA) and Duncan's multiple range test were carried out to determine significant differences (*p* < 0.05) between the means by SPSS 16.0 program (Chicago, IL, USA).

## 5. Conclusions

This study described the chemical compositions and antimicrobial properties of cardamom, cumin, and dill weed EOs, as well as their mechanism of action against *Campylobacter* spp. The results indicated that tested EOs were effective inhibitors by directly acting through membrane integrity of both *C. jejuni* and *C. coli*. Based on the current study, we introduce that using tested EOs will help to control the diseases caused by *Campylobacter* spp. However, in addition to in vitro experiments, in vivo studies are also required. Moreover, further studies are needed to balance between the sensory acceptability and antimicrobial activity.

## Figures and Tables

**Table 1:** Chemical compositions of essential oils.

No	Compounds ^a^	RI ^b^	RI ^c^	Peak Area ^d^ (%)		
				Cardamom	Cumin	Dill Weed
1	α-Pinene	1032 ^1^	1033	1.3 ± 0.0	0.7 ± 0.0	1.0 ± 0.0
2	β-Pinene	1118 ^1^	1124	-	14.4 ± 0.0	1.4 ± 0.0
3	Sabinene	1132 ^1^	1134	4.3 ± 0.0	0.5 ± 0.0	-
4	Myrcene	1174 ^1^	1173	0.8 ± 0.0	0.8 ± 0.0	-
5	α-Phellandrene	1176 ^1^	1178	-	0.5 ± 0.0	7.4 ± 0.0
6	Limonene	1203 ^1^	1211	2.1 ± 0.0	-	27.4 ± 0.1
7	1,8-Cineole	1213 ^1^	1222	29.2 ± 0.1	-	-
8	β-Phellandrene	1118 ^1^	1224	-	-	1.8 ± 0.0
9	γ-Terpinene	1255 ^1^	1264	-	16.9 ± 0.0	-
10	*p*-Cymene	1280 ^1^	1287	-	8.3 ± 0.0	4.7 ± 0.0
11	*trans*-Sabinene hydrate	1474 ^1^	1475	0.5 ± 0.0	-	-
12	Dill ether	1529 ^2^	1542	-	-	9.2 ± 0.0
13	Linalool	1553 ^1^	1553	3.6 ± 0.0	-	-
14	Linalyl acetate	1565 ^1^	1569	5.7 ± 0.0	-	-
15	Terpinen-4-ol	1611 ^1^	1621	0.6 ± 0.0	-	-
16	*trans*-Dihydrocarvone	1624 ^1^	1637	-	-	0.5 ± 0.0
17	*cis*-Isodihydrocarvone	1645 ^2^	1658	-	-	0.9 ± 0.0
15	α-Terpinyl acetate	1706 ^3^	1727	43.4 ± 0.1	-	-
16	Geranyl acetate	1765 ^1^	1769	0.8 ± 0.0	-	-
17	Carvone	1751 ^1^	1774	-	-	41.6 ± 0.1
18	Cumin aldehyde	1802 ^1^	1823	-	24.1 ± 0.1	-
19	*p*-Mentha-1,3-dien-7-al	1811 ^4^	1838		26.7 ± 0.1	-
20	Geraniol	1857 ^1^	1855	0.7 ± 0.0	-	-
21	(E)-Nerolidol	2050 ^5^	2048	0.9 ± 0.0	-	-
22	Cumin alcohol	2113 ^1^	2127	-	0.6 ± 0.0	-
	Total			94.0 ± 0.2	93.9 ± 0.0	95.8 ± 0.2

^a^ Identification based on the comparison of mass spectra and co-injection with standard Alkan series (C_7_*–*C_40_), ^b^ Retention indices from literature [24] ^1^, [25] ^2^, [26] ^3^, [27] ^4^, [28] ^5^, ^c^ Retention indices relative to standard Alkan series (C_7_*–*C_40_), ^d^ Peak area (± SEM) was obtained by averaging three different determinations obtained by GC-FID, -: Not detected.

**Table 2:** DIZ, MIC, and MBC of essential oils against *Campylobacter* spp.

	*C. jejuni*			*C. coli*		
	DIZ ^1^ (mm)	MIC ^2^ (µL/mL)	MBC ^3^ (µL/mL)	DIZ (mm)	MIC (µL/mL)	MBC (µL/mL)
Cardamom	24.75 ± 2.00 ^c^	0.025	0.025	25.58 ± 2.23 ^c^	0.025	0.025
Cumin	19.75 ± 2.70 ^a^	0.050	0.050	21.08 ± 1.38 ^a^	0.050	0.050
Dill weed	22.25 ± 1.60 ^b^	0.025	0.025	23.33 ± 2.57 ^b^	0.012	0.012

^1^ DIZ: Diameter of inhibition zone, ^2^ MIC: Minimum inhibition concentration, ^3^ MBC: Minimum bactericide concentration, ^a^^–^^c^: Different letters within each column indicate statistically significant differences between the means (*p* < 0.05).

**Table 3:** Cell constituent release of *Campylobacter* spp. after adding essential oils.

Essential Oil	Concentration	Cell Constituent Release (OD_260_) ^1^	
		*C. jejuni*	*C. coli*
Cardamom	Control	0.071 ± 0.014 ^a^	0.022 ± 0.018 ^a^
	MIC ^2^	0.158 ± 0.009 ^b^	0.106 ± 0.017 ^b^
	2× MIC	0.201 ± 0.018 ^c^	0.201 ± 0.019 ^c^
Cumin	Control	0.059 ± 0.033 ^a^	0.047 ± 0.002 ^a^
	MIC	0.173 ± 0.009 ^b^	0.122 ± 0.022 ^b^
	2× MIC	0.282 ± 0.090 ^c^	0.205 ± 0.021 ^c^
Dill weed	Control	0.033 ± 0.005 ^a^	0.054 ± 0.007 ^a^
	MIC	0.175 ± 0.011 ^b^	0.133 ± 0.007 ^b^
	2× MIC	0.280 ± 0.013 ^c^	0.252 ± 0.008 ^c^

^1^ OD_260_: optical density at 260 nm, ^2^ MIC: Minimum inhibition concentration, ^a^^–^^c^: Different letters within each column indicate statistically significant differences between the means (*p* < 0.05).
